# Adolescents’ Perceptions of Gender Aspects in a Virtual-Reality-Based Alcohol-Prevention Tool: A Focus Group Study

**DOI:** 10.3390/ijerph19095265

**Published:** 2022-04-26

**Authors:** Christina Prediger, Robert Hrynyschyn, Iasmina Iepan, Christiane Stock

**Affiliations:** 1 Charité–Universitätsmedizin Berlin, Corporate Member of Freie Universität Berlin and Humboldt-Universität zu Berlin, Institute of Health and Nursing Science, Augustenburger Platz 1, 13353 Berlin, Germany; christina.prediger@charite.de (C.P.); robert.hrynyschyn@charite.de (R.H.); iasmina.iepan@charite.de (I.I.); 2 Unit for Health Promotion Research, University of Southern Denmark, Degnevej 14, 6705 Esbjerg, Denmark

**Keywords:** gender, virtual reality, alcohol prevention, adolescents, focus groups, thematic analysis

## Abstract

Virtual reality (VR) is an innovative tool for alcohol prevention among adolescents. However, many aspects of virtual simulations for alcohol prevention remained unstudied, and research on opportunities for tailoring such tools to users’ gender using avatar-based pathways is lacking. The present study, therefore, explores adolescents’ perceptions of gender portrayal and gender tailoring using *Virtual LimitLab*—a VR simulation for building refusal skills for dealing with peer pressure to consume alcohol. Focus groups were conducted after individual simulation testing with 13 adolescents in four groups, whose statements and discussion underwent thematic analysis. Three main themes were identified: the *relevance of gender*, opinions on different *tailoring options* for gender, and opinions on *flirt orientation*. Divergent arguments for different tailoring options and representations of gender in the simulation were proposed. Some participants changed opinions during discussions. Sexual harassment was consensually deemed an important issue that is linked to both partying and alcohol and was concluded to require being addressed along with alcohol prevention. A consensus also formed around open flirting possibilities (regardless of gender), and awareness of non-binary peers was raised. Based on the observed sensitivity of the adolescents to gender diversity, it is necessary to include LGBTQIA+ adolescents when developing gender-sensitive simulations.

## 1. Introduction

Alcohol is one of the most commonly used and misused substances among adolescents in Europe [1]. Prevention in this target group is crucial because the regular and early use of alcohol increases the likelihood of problematic consumption and dependency in later life [2,3,4]. Early intervention is important because attitudes, drinking motives, and consumption patterns are often formed during adolescence [3].

While alcohol consumption by adolescents in European countries has been decreasing slightly in recent years [1], the overall prevalence remains high. Indeed, the latest data for Europe revealed a lifetime prevalence of alcohol consumption of 79% among 15–16-year-olds, with about one-third (34%) of adolescents having participated in binge drinking within the previous 30 days [1]. In Germany, the prevalence of lifetime alcohol consumption was even higher: 90% among 15–16-year-olds, with 54% having taken part in binge drinking at least once within the previous 30 days—one of the highest country-specific rates in Europe [1].

In terms of gender, boys in almost all European countries report more problematic alcohol consumption than girls: They begin drinking alcohol earlier than girls (36% of boys have tried alcohol by age 13 or younger compared with 29% of girls), and they display a higher prevalence of both binge drinking (36% for boys compared with 34% for girls) and intoxication at an early age (8% for boys compared with 5% for girls) [1]. Boys who have drunk within the previous month have done so more frequently and report having consumed higher volumes than girls [1]. However, time trends revealed a narrowing of the gender gap in binge drinking, with rising rates among girls in recent decades. Meanwhile, some indicators—such as lifetime use (79% for boys compared with 78% for girls), 30-day use (47% for boys compared with 46% for girls), and intoxication rates within the previous 30 days (14% for boys compared with 13% for girls)—do not differ much between girls and boys [1]. These findings are in line with national data from Germany, which revealed higher rates of alcohol consumption among boys than girls but show significant differences only in the regular use of alcohol and in binge drinking among 12–17-year-old boys compared with girls [4].

Given the existing differences in high-risk alcohol use, gender-specific interventions for girls and boys have been suggested [5,6]. A review by Dir et al. [5] on adolescents’ binge drinking concluded that girls might be more sensitive to peer pressure, while for boys, the stereotype that binge drinking is masculine may be a more important risk factor. Other research has revealed that in terms of alcohol intoxication, peer pressure has a stronger impact on boys [6]. Additionally, overestimation of peer alcohol use serves as a risk factor for disruptive drinking among boys [7]. Overall, research shows that tailoring prevention programmes to the specific characteristics and needs of adolescents is a key aspect of the effectiveness of such programmes [8]. Therefore, more research on gender-specific needs for alcohol prevention is needed [5].

Virtual reality (VR) is an innovative medium for alcohol prevention. As VR provides a digital 3D simulated experience that is made tangible via special software and hardware [9], it can stimulate learning through increased immersion and interactivity compared with traditional learning methodologies [10]. Moreover, social interactions can be simulated, and behavioural options can be trained in a safe environment [11]. VR also offers good opportunities for tailoring: by altering game characters (avatars), it is possible to design specific pathways and scenarios that are tailored to different diversity characteristics, such as gender. As gender-tailored interventions are promising tools for stimulating behavioural changes [12,13,14], this feature in VR has potential in the context of alcohol prevention.

Only a few interventions exist that use VR for alcohol prevention among adolescents [15]. Since 2017, two simulations have been developed, namely the VR component of the Australian alcohol education program *Blurred Minds* and the Danish *VR FestLab* application. These simulations were created as refusal skills training to resist peer pressure regarding alcohol consumption. Studies on user perceptions of the existing VR tools for alcohol prevention show promising results for VR in this context [16,17,18,19]. A cluster-randomised controlled trial investigated the effectiveness of the *VR FestLab* application in terms of increased competence in dealing with alcohol and peer pressure but did not show a significant increase in adolescents’ self-efficacy expectations to refuse alcohol [20]. The reasons for this could be either in the study design (e.g., lack of power and/or too short gameplay as intervention) or deficiencies in the educational elements of the application, so further research to improve effectiveness is necessary.

Despite gender-specific conceptualisations of VR simulations in this field, considerations regarding gender have not been outlined in publications thus far [15]. As gender-sensitive and diversity-oriented perspectives are advised for digital health promotion [21], further research that examines the opportunities and challenges of tailoring VR simulations according to gender is needed. While it is possible to tailor interventions that correspond more accurately to the realities that girls and boys experience and that are thus more effective, there is also a risk of (re-)manifesting and consolidating dichotomies from the categories of female and male and thus also of reproducing and reinforcing stereotypes [22]. In addition, this could lead to inappropriate homogenising of within-gender-group differences and could also exclude all those who do not (only) identify with one of the two options of female or male, thereby not capturing gender diversity [16]. In short, the challenge is to use gender as a category of difference and analysis in order to create realistic and relatable interventions that do not reinforce difference-theoretical pre-assumptions or exclude certain groups. By taking an intersectional perspective in public health into account [23], an intra-categorial approach [24] could be applied that explores one category (here, gender) by focusing on differences and complexity within the category. Only subsequently would the perspective be broadened to include further categories of difference (e.g., cultural or economic background, ability, etc.) and their intersection with one another. Gender is understood here as a sociocultural variable that complements sex as a biological variable [25] and that comprises more than two possible expressions, thereby containing diversity as well as within-group heterogeneity. Moreover, both gender and sex are understood multidimensionally [26] and in dynamic interaction across the lifespan in terms of their impact on health. Throughout this text, the terms “woman/man”, “female/male”, and “girl/boy” (and their plurals) are all used to refer to gender.

The present study therefore aims to explore and investigate adolescents’ perceptions of gender aspects and gender-tailoring options in a VR-based alcohol-prevention tool.

## 2. Materials and Methods

### 2.1. Research Design

Due to the explorative character of the study, a qualitative study design was chosen. To aid in understanding adolescents’ perceptions, opinions, and thoughts on gender in the described *Virtual LimitLab* VR simulation, focus groups were conducted after the adolescents had tested the prototype. Focus groups were chosen as they are suitable for effectively collecting a range of different positions and thoughts [27] and because the appropriateness of individual opinions and statements can also be assessed via feedback from the group through participants’ agreement and disagreement with statements made by other group members [28]. Transcripts were analysed using the thematic analysis of Braun and Clarke [29]. The study is in line with the quality criteria of qualitative research [30] and with the consolidated criteria for reporting such designs [31].

### 2.2. Virtual LimitLab

The tested alcohol-prevention tool *Virtual LimitLab* is a 360° filmed house-party simulation for adolescents that was designed to train their drinking-refusal self-efficacy in order to enable them to better resist the peer pressure to consume alcohol. Different types of VR can be differentiated according to the degree of interaction possibilities [32]. As a 360° video, *Virtual LimitLab* can be classified as a training world that is shaped by the viewpoint of the camera and that offers limited ability to move freely. Nevertheless, interactivity is provided by the plot, which changes according to the user’s choices at the end of each scene. *Virtual LimitLab* is a prototype version of the Danish simulation *VR FestLab* that has been overdubbed in German. For more details on the participatory development of *VR FestLab*, see Lyk et al. [33] and Vallentin-Holbech et al. [17].

In the *Virtual LimitLab* starting scene, the player must choose a gender (female or male, as represented by the bathroom figures). After a pre-party scene, the player attends a house party of adolescents and can enter different scenes (e.g., the bar, the dance floor, a beer-pong table). After each take, the player is confronted with different behavioural choices. Options include choosing alcoholic or non-alcoholic drinks when peers encourage the user to drink. Further behavioural options include dancing, flirting, playing a game, or supporting drunk peers. Depending on the player’s individual choices and blood-alcohol concentration (BAC), the subsequent plot changes. A few scenes also change according to the chosen gender. BAC is calculated based on the number of alcoholic drinks consumed, the body mass index (BMI) of the average 16-year-old girl or boy, and the time. Navigation is possible via head motion, and selections are made by staring at option buttons. Positive and negative role models for alcohol consumption are presented in the different scenes. Social feedback is given by direct peer reactions and by mobile text messages that the user receives in the last scene on the morning after the party. Positive experiences include flirting, having a conversation with the older bartender, and receiving positive feedback in the text messages the following day. The social costs of excessive alcohol consumption are demonstrated by negative consequences, such as not being able to flirt, vomiting, blacking out due to intoxication, and receiving negative feedback the morning after. The aim of the simulation is for the user to learn how to deal with peer-pressure situations and to consider consuming alcohol more moderately. For detailed results on the effectiveness of the Danish version—*VR FestLab*—in terms of user experience and drinking-refusal self-efficacy, see Guldager et al. [19,20].

### 2.3. Inclusion Criteria and Recruitment

Corresponding with the intervention’s target group, inclusion criterion for participation was an age of 15–18 years. Participants are referred to as “adolescents” and therefore also as “girls” and “boys”, even though the age of 18 is legally defined as adulthood in Germany. A further criterion was the self-reported ability to participate in a focus group discussion in German language. No further exclusion criteria were set. As a pilot test of the German version *Virtual LimitLab,* a non-probabilistic convenience sample was chosen. Youth organisations and youth welfare institutions in Berlin were contacted via mass email (n = 337) and telephone (n = 16). These organisations included, for example, youth clubs, cafés, centres, sports clubs, music schools, open social work, and local initiatives covering a wide range of after-school offers for adolescents. This variety was purposive to avoid sending out invitations to certain subgroups of adolescents. Additionally, snowball recruitment was used by encouraging participants to share the study invitation with friends. Adolescents who were interested and who had submitted informed-consent forms were invited to focus group meetings.

### 2.4. Data Collection and Processing

Data collection consisted of focus groups and a short questionnaire. In total, four focus groups (A–D) with four participants each were scheduled for August 2021 at the Institute of Health and Nursing Science, Charité–Universitätsmedizin Berlin.

As homogeneity in group composition is advised in focus group design [34], the groups were designed to be gender homogenous for girls and boys in order to create a shared background of gender topics. After a welcoming round and the possibility to ask questions, a group game was initially carried out to strengthen bonding [34]. After this introductory phase, each participant tested the VR simulation individually for at least 20 min, which is sufficient for one or two playthroughs. This testing was used to stimulate subsequent focus group discussion. Each participant was handed a head-mounted display (Destek V5, Thinkline Technology Ltd., London, UK) with a smartphone inside (Samsung Galaxy A21 devices, Samsung, Suwon, Korea), on which the simulation was installed. The data collection period was limited to two weeks, for which technical devices were rented and prepared.

A semi-structured interview guide with open-ended questions was used to ensure that topics of interest for the research question were covered and handled flexibly according to the context of each focus group so that new topics of relevance could be discussed. The interview guide consisted of five sections on the perception of gender within the simulation and could be discussed in flexible order. These sections were

1. Perceptions about the simulation;
2. Assessment;
3. Development needs;
4. Suggestions for improvement;
5. Potentially important categories for tailoring other than gender.

This interview guide was developed based on Helfferich’s [35] four steps for interview guides (collection, consideration, sorting, and subsuming). All participants were previously unknown to the researchers. Two interviewers (C.P. and R.H.) were present—one male and one female, who both have had experience in conducting qualitative interviews. Both interviewers introduced themselves as researchers and explained the scope of the study. Additionally, a third, female researcher assistant (I.I.) was introduced as a colleague who would take down field notes and support the preparation and conducting of the focus groups. Field notes were written after each focus group and included general observations and ideas. The duration of each group was set to 30 min.

The focus groups were audio-recorded, transcribed verbatim, and pseudonymised prior to data analysis. The short questionnaire collected information from each participant on their age, the type of school they were attending, and whether they have had experience with alcohol and with VR.

### 2.5. Data Analysis

The six steps for thematic analysis described by Braun and Clarke [29] were used. Thematic analysis can be used for “identifying, analysing and reporting patterns (themes) within data” [29] (p. 79) and can be deductive, inductive, or both in addition to being iterative, and ongoing analysis can thus include coming back to previous steps during the process. The advantage of this approach is its flexibility, which is precisely why researchers need to outline in detail exactly how they apply and execute this approach in their research, which provides clarity on their process and practice [29]. The following decisions about analysing gender aspects were made:

Themes were defined as meaningful patterns on a higher level and consisted of codes that were strongly related to one another and were defined by importance and not by the quantity of the quotes. The analysis focused on providing a detailed account of one aspect—namely perceptions and statements on gender in the context of the VR simulation. Results were collected on the semantic surface, and no focus was placed on participants’ deep-seated beliefs because the research question involved explicit perceptions. Due to the deductive–inductive openness of the method, deductive candidates for codes based on the interview guide were conceived but retained for an inductive coding. However, the content of the transcripts was shaped by the interview guide and the research question, and deductive code assumptions could thus be verified by inductive coding. Nevertheless, codes—and especially sub-codes—were formed inductively. In greater detail, the thematic analysis consisted of the following six steps:

(1) Familiarisation with the data: Initially, transcripts of the focus groups were re-heard, re-read, and corrected where necessary, and first ideas were noted down. (2) Generation of initial codes: Afterwards, initial codes were formed inductively by marking text passages broadly while keeping contradictions, the context, and double occupancy in the passages. MAXQDA software (2014) (VERBI Software GmbH, Berlin, Germany) was used for computer-assisted organisation and analysis. These two steps were performed by one researcher. The subsequent steps were each discussed in the research team. (3) Search for themes: Theme candidates were abstracted by organising codes on a higher level in terms of shared topics, strong relation, or content coherency. (4) Review of themes: Subsequently, these themes were reviewed by checking the internal homogeneity and external heterogeneity in relation to the coded extracts. Therefore, all transcripts were re-read, and for some passages, either re-coding was performed, or codes were combined or split where necessary. To aid in understanding, a graphical map was generated for visualising the relation of the codes to one another. (5) Defining and naming themes: Theme and code candidates were checked once again in an iterative process by generating definitions and relations to one another. A list of themes and codes was written and used when going through the material one last time and for analysing the dataset once more. (6) Production of the report: Finally, to prepare the present article, themes and codes were ordered on a list, which allowed them to be shaped into an overall story that could be comprehensibly presented to readers. The presentation order does not represent a hierarchy but rather a logical plot that allows the presentation and interpretation to be easily followed. The list was then filled with all corresponding statements for each code and served as the basis for writing the present article. Discussion among the research team (C.P., R.H., I.I. and C.S.) and reflection with an external qualitative research group (Qualitative Forschungswerkstatt, Charité) were used to consider and refine the analysis and interpretation in order to meet the quality criterion of inter-subject comprehensibility [30].

### 2.6. Ethics

Ethical approval was obtained from the ethics committee of the Charité–Universitätsmedizin Berlin (file number EA2/154/21). Participation was voluntary and written as well as oral information on the study was provided. Informed-consent forms were distributed at least three days before participation. For participants under age 18 years, the consent of their parents or legal guardians was also obtained. Furthermore, participants gave informed consent that the focus groups could be digitally recorded. Personal data were processed in accordance with both the European General Data Protection Regulation and the Declaration of Helsinki. All participants provided informed consent for taking part in the study. After the study had been concluded, the participants each received a voucher as an expense allowance worth €30.

## 3. Results

In total, 13 adolescents participated in four focus groups. As shown in Table 1, the age varied from between 15 and 18 years, with a mean of 16 years. One of the participants claimed to not have had any experience with drinking alcohol (one out of 13). Most participants (10 out of 13) had had experience with VR. Most participants were attending Gymnasium (German upper-level secondary school or grammar school) (11 out of 13), and two were attending Gesamtschule (German integrated comprehensive secondary school). Focus groups were recruited gender-homogenously for girls and boys. In total, three focus groups with girls and one group with boys took place.

**Figure 1:**
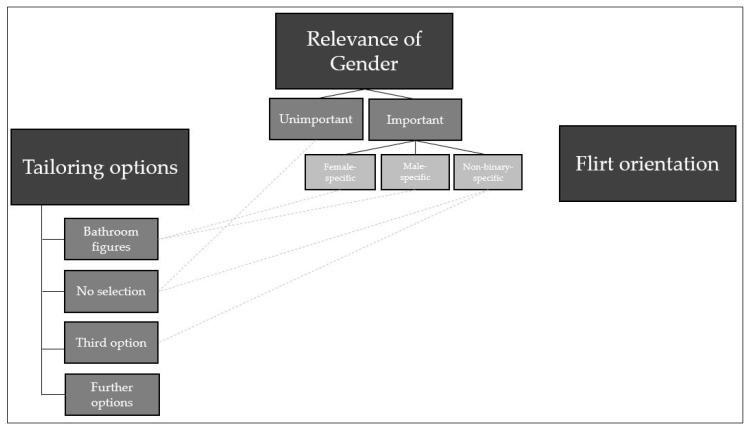
Overview of the final thematic map.

Following the thematic analysis, a thematic map was generated. Figure 1 provides an overview of the final themes, codes, and sub-codes. The analysis identified three themes:

1. Relevance of gender;
2. Tailoring options;
3. Flirt orientation.

While the first theme—*relevance of gender*—comprised both identity and orientational aspects, *tailoring options* and *flirt orientation* focused only on identity and on aspects of gender orientation, respectively.

The theme *relevance of gender* further consisted of two codes: *unimportant* and *important* relevance of gender in the context of alcohol and partying. The code *important* was then divided into further sub-codes of statements on the *female*, *male*, and *non-binary*-*specific* importance of gender. The second theme—*tailoring options*—consisted of gender-identity topics that can be represented in VR by avatars. The theme comprised the codes of *bathroom figures*, *no selection*, *third option*, and *further options* for tailoring. Some of these codes were connected to sub-codes of the first theme—*relevance of gender*—because they represented the consequences of the respective relevance of gender in this simulation. The third theme—*flirt orientation*—consisted of statements on orientation; it was not further divided and was not connected to the other (sub-)codes. A further description of codes and sub-codes is presented along with at least one illustrative quote for each (sub-)code in the following section. To fulfil the quality criterion of empirical foundation [30], further example quotes that support the results are indexed in brackets and can be found in the Supplementary Materials (Table S1).

General results on group dynamics and comparisons between the girls’ and boys’ focus groups are based on field notes. Controversial discussions took place in all groups. In comparison with participants in the boys’ group, participants in the girls’ groups tended to be more consensus-oriented and were more likely to change their opinions during discussions. While opinions in the girls’ groups were formed dynamically through group interaction, individual statements within the boys’ group were more distinct. In addition, the participants in the boys’ group showed less openness to changing their views throughout group discussions as they did not try to agree with one another, and opposing positions thus tended to persist.

### 3.1. Theme 1: Relevance of Gender

The relevance of gender for alcohol prevention was not ascertained with a direct question. Nevertheless, the topic was addressed by participants within many phases of the focus groups. In general, this theme is divided into approving and disapproving statements about either the VR simulation or a party context in the adolescents’ real lives. Arguments were accumulated in the respective codes of *unimportant* and *important* relevance of gender.

The code of unimportant comprises statements referring to gender as “irrelevant” in the VR simulation. In multiple statements, participants denied the relevance of gender in the simulation, as illustrated in the following statement: “*So, gender doesn’t really play a role [in the simulation]*” (C1) (for further selected example quotes (1,2), see online Supplementary Table S1). In addition, participants stated that they had not noticed gender differences in the simulation (3,4), even after the avatar of the other gender had been selected in a second playthrough (5). This finding is reflected in the following statement: “*Yeah, so I have... I wanted to be the other one [avatar] once to see if it [the simulation] was completely different or something... but it is apparently not*” (B4). Some of the participants even forgot which gender avatar they had chosen during testing (6,7), which the participants explained by stating that players cannot see themselves in the VR simulation (8,9) and that both interactions with the simulation characters and the language appeared to be gender-neutral (10,11). In this regard, many of the participants suggested or agreed with the notion of completely leaving out the gender selection at the start of the simulation. This idea is explored further below under the code of *no selection*. According to the participants, possible reasons for the irrelevance of gender in the simulation included the lesser importance of gender for the younger generation (12) and the notion that the individual person rather than the gender identity was the relevant characteristic in the simulation (13), as represented in the following statement: “*So, I don’t think it’s the gender that is really relevant, but the person*” (C1).

In contrast, among the code of *important*, gender was declared to be relevant for the simulation (14–16) and especially for party situations in real life (17). Participants were asked whether and to what extent the simulation was perceived to realistically reflect female and male experiences and how it could reflect a gender-specific experience. The code was then divided into further sub-codes based on the *female*, *male*, and *non-binary* gender-specific relevance of gender. One argument for the relevance of gender in the simulation was that it was possible to identify when the simulation reflected gender-specific party experiences, as highlighted by the following statement: “*So, I would say, ideally, it [gender] is really there. I think otherwise, you also have the problem that you can no longer identify with it [the avatar] very well as a man or as a woman*” (B2). While some of the participants denied the relevance of gender within the simulation, the relevance of gender for real party situations was stated to indeed matter (17). One participant mentioned that at a real party, the experience would be “*really completely different depending on gender*” (B3). When examples were asked for, gender differences were stated to be important for the simulation, especially in verbal expressions with one’s peers (15,16).

The discussion on gender-specific experiences in the context of partying and alcohol consumption led the participants to make statements on *female-specific* experiences as follows: Three of the four focus groups broached the topic of sexual harassment without being prompted when potential gender differences were asked for. In two groups, this topic was first mentioned when gender-specific differences were asked about. Sexual harassment was always mentioned by the girls and boys alike as an issue that women are forced to deal with. Women were viewed as the targets of unwanted approaches in a party context (18,19), whereas men were not (20,21). A depiction of sexual harassment in the simulation was viewed as desirable (22,23) as it would add to the realism of the portrayed party (24). Participants concluded that girls should encounter this element in the simulation in order to learn skills that could help them navigate similar situations in real life, as the following statement illustrates: “*I think that it [the simulation] could be more offensive towards women. If I—as a woman—want to [learn to] deal with the subject [of sexual harassment], I really want to be prepared for this kind of risk.*” (A2). The current simulation was perceived in this regard as being still “*harmless*” (C2) or “*pleasant*” (C2). One idea for adding sexual harassment was to conceptualise the simulation such that the shorter the chosen outfit of a female character was, the more sexual harassment the player would experience (25). This idea was rejected by another participant in a further discussion for being problematic and sending “*the wrong message*” (A1). Divergent opinions arose regarding how to portray sexual harassment in the simulation. Such scenes were viewed as being potentially inappropriate for certain age groups (26). The portrayal of this theme was also regarded as a potential deterrent from the goal of preventing adolescents from engaging in binge drinking (27), and one participant questioned how an adequate representation of sexual harassment could be realised (28). Upon further discussion and reflection, the participants changed in the view that sexual harassment should be exclusively experienced by the female character (29,30). Portraying sexual harassment only against women was stated to likely perpetuate stereotypes and to only strengthen the stigma for men who experience it. A more inclusive approach to the topic that does not conceptualise sexual harassment as an issue that only women have to deal with was then favoured as being more appropriate. Additionally, ideas such as including female-typical verbal expressions (31), a gender symbol within the visual field to remind the user of the chosen gender of the avatar (32) or visible gender-typical clothing (33) were brought up in one of the girls’ focus groups.

Correspondingly, according to the participants, a typical male experience at a party involves *male-specific* verbal utterances, especially among boys (34). In contrast to female-specific topics, male-specific topics were brought up less in the discussions. Regarding the broadly discussed topic of sexual harassment, boys were seen as not having to contend with unwanted approaches, as reflected in the following statement: “*You… you have to worry much less [as a boy]. So, at a party, then, somehow, a girl has to worry*” (B4). Furthermore, a male-specific scene was discussed in which the player is rejected when flirting with a male character in the simulation after having chosen a male avatar and as a reply hears the statement “Hey bro, I’m not gay. But we can have a beer.” That reaction was perceived by one participant who experienced this scene to be mean but to nevertheless be a good male-specific depiction of real life for the simulation (35).

The relevance of gender was not only discussed in terms of girls and boys but also brought up in terms of peers who self-identify as a gender other than female or male. In their statements on this topic, none of the participants referred to themselves; instead, they referred to other peers they knew or could imagine (36). Therefore, the statements among this sub-code are conjectures. In three of the four groups, the topic was raised by participants, for example, with the question “*Is there also something that already exists for non-binary or transsexual people?*” (A3), and the topic was then further discussed. Firstly, participants expressed that a binary conceptualisation of gender in the simulation might be problematic and less realistic (37) for some of their peers. Reasons included the notion that it is necessary to be inclusive of non-binary peers (36,37), as reflected in the following quote: “*If I were a non-binary person, it would be nice to see that I exist, that I am not immediately reminded, so to speak, that in society, only men and women are accepted*” (B2). In the three groups in which the topic came up, the discussion resulted in different tailoring options (e.g., to leave out gender selection at the start or to enter a third option), which are exemplified among these sub-codes further below. An additional idea for better representing gender diversity was to include a non-binary character among the characters in the simulation, which was thought to add to the diversity but to also have the potential to be irritating, as the following statement illustrates:

*“And maybe you could make the person on the couch really... um... non-binary and then somehow bring in a second, a third person who really also obviously... maybe that would bring in a bit more diversity, but... but of course, it can also be a bit annoying for people who don’t... well, who are just really... who are straight and all. I don’t know.”* (C2)

On the other hand, participants found the relevance of non-binarity to be less important for several reasons: firstly, because the avatar choice at the beginning would be a free choice (38), and secondly, because in reality, it would be seen as “normal” to be classified as female or male (39). Furthermore, one participant questioned the relevance of addressing non-binarity in this context because only a small number of non-binary people was assumed to exist (40).

### 3.2. Theme 2: Tailoring Options

The second identified theme comprises statements on possible *tailoring options*. Perceptions, opinions, and thoughts on the current design of *Virtual LimitLab*—in which a female or male avatar has to be chosen—were directly asked for. Further possibilities for tailoring were also discussed. Each code represents possible identity options that are linked to the stated (ir)relevance of gender in the context both of the simulation and of a real-life party situation.

The representation of tailoring options with female and male *bathroom figures* at the start of the simulation was perceived differently by the participants. Initially, the binary choice did not raise any point of discussion (41,42). On the one hand, when asked about the use of bathroom figures as symbols for female and male, most participants initially rated it as “*good*” (A2, D1), “*understandable*” (B3), and “*sufficient*” (D3). On the other hand, other participants stated that they did not like the symbols, as for example in the following statement*:* “*Please, no stick figures …, [but] at least good [that] you don’t use colours*” (A3). Some participants changed their opinion or agreed upon discussion that a binary choice might not be up to date, as was the case with the following participant:

*“But now that we’re talking about it, I’d also like it better if it [the simulation] were adapted so that it appealed to everyone... so that you didn’t just have the choice of male and female because it’s… I think it’s just not the current state of our society.”* (A1)

Pro-arguments for a binary version of gender in the simulation included the possibility to design scenes as female- or male-specific (43). This link is represented in the thematic map by a connecting line to the sub-codes of female- and male-specific. Additionally, it was stated that the choice is a free option, that it does not necessarily need to correspond to what someone identifies with (44), and that alternatives with more than two gender options might not be able to be realised (45). Contra-bathroom-figure arguments included the notion that just because one person feels addressed does not mean everyone feels addressed (46). Furthermore, it was stated that the simulation was perceived as mainly gender-neutral (47), and therefore, in most groups, the idea came up to leave out the choice of gender altogether, which will be enfolded in detail under the following code.

The code of *no selection* comprises statements and thoughts about leaving out the choice of avatar’s gender entirely. As *no selection* was stated to be a possible solution for gender diversity and as participants stated that gender did not change the simulation much, this code is related to the sub-codes of *non-binary-specific* and *unimportant*. Many participants suggested or voted after discussion for this variant and described it as the “*best*” (A4, C1) option and as a “*direct*” (C1) and “*objective*” (C1) way to enter the simulation in which the player only has to worry about being the player (48–50). No contra-arguments were directly given on the suggestion to leave out the choice of avatar gender at the beginning. In contrast, mentioned justifications for leaving out the avatar gender selection at the beginning included the idea (1) that nothing would be missing without it (51), (2) that the simulation does not remarkably change depending on which avatar is chosen (52,53), (3), that leaving the decision out could be more pleasant for those who do not identify as a girl or a boy (54), and (4) that the challenge and complexity of addressing everyone’s gender in the right way can be avoided (55), as illustrated by the following statement:

*“I generally don’t think that you really need a selection because if you actively do not feel that it makes much of a difference, then I think it’s just more complicated to do that now… to make a selection and to address everyone with it.“* (A1)

Another solution that was brought up and discussed was to create a *third option* in addition to girl and boy. Opinions differed on this idea during discussions. While a third category was suggested and rated as good and possible on the one hand (56,57), other participants did not see a need for it (58) or even rated the idea as “*counterproductive*” (B3) on the other hand. Those who rejected a third option did so for different reasons—namely due to the notion (1) that for one participant, the social dimension of gender was based on the assumption that there are only two biological sexes (59), (2) that regardless as to whether non-binarity exists, the common perception at a party would be binary (60), (3) that the female or male options are both available to participants (61), and (4) that the simulation was designed to be mostly neutral and a third option would thus not change the experience (62). Pro-reasons for including a third option consisted of the notion that some adolescents might be offended by the existence of only two gender options (63) and that a third gender possibility could be a “*nice*” (B2) option for non-binary peers, as one participant explained:

*“I think it [a third option] could be added. It wouldn’t change very much, but it would be nice, if I were a non-binary person, to see that I’m there, that I’m not immediately reminded that in society, only men and women are accepted. Otherwise, yes, I don’t think you have to change so much in the game, but at least you would have the option.”* (B2)

Possible labels for a third option were asked for, resulting in hypotheticals such as “*neutral*” (D2), “*none of the above*” (D3), “*irrelevant*” (D1), and “*non-binary*” (C2, B1). Additionally, it was stated that in the German-speaking context, currently, there would be no good equivalent to the English word “non-binary”, and it should be waited until there would be a generally understandable term (64).

Furthermore, the interviewer brought up further options for possible tailoring and asked the participants for their opinions. The participants were asked about the possibility to customise their avatar by constructing an individual look (e.g., hair colour, glasses, etc.). On the one hand, this idea was met with excitement by some participants as this type of tailoring was viewed as especially effective in reflecting the individuality of each player (65). On the other hand, a detailed customisation process was viewed as “*laborious*” (A4) and “*long*” (B2). One participant raised the concern that such a feature would distract from the purpose of the game (66), while another questioned whether tailoring the appearance of the avatar would be reasonable in a game that has a short playtime (67). Participants also proved receptive to the idea only if it would result in in-game consequences (68). Customisation without any influence on the gameplay was viewed as “*superfluous*” (A1).

Another refined tailoring opportunity that was asked about involved the BAC bar, which is visible during the simulation. Participants mentioned that the current calculation—based on the average body mass index of a 16-year-old girl or boy—could be misleading (69) and could even create a harmful underestimation (70) of the concentration among users who deviate from the average. The option to enter the player’s exact height and weight was suggested as an opportunity to tailor the simulation to the actual physicality of each player (71), which was regarded as “*interesting*” (D3) and “*quite a good idea*” (B3) in the boys’ focus group. In the girls’ focus groups, concerns were raised because entering such sensitive data could be deemed “*unpleasant*” (D3) and could also be lied about (72,73). As a compromise, one participant suggested that the simulation could explain how the BAC is calculated at the beginning and include the opportunity to individualise the calculations by entering personal values, if desired (74). In the girls’ groups, the desire to include an explanation of BAC was also mentioned (75).

Additionally, participants were asked if variables or characteristics other than gender were important when tailoring the simulation. Some participants expressed the idea of tailoring according to sexual orientation, as this design would protect players from being approached by the undesired gender (76,77). However, other participants replied that it is okay to be approached by anyone if the player has the opportunity to decline advances and to express a lack of interest. No further variable that was important for tailoring was mentioned when participants were asked. In this context, some participants stated that the characters in the simulation could be more diverse (78,79). Aside from non-binary characters among the actors, examples included characters who cannot speak German perfectly or with an accent (80,81) and “*dark-skinned people*” (82,83).

Bringing the discussion back to tailoring, one participant mentioned that the most effective form of tailoring could take place through the player’s actual behaviour and choices at the simulated party (84,85): “*I think personalisation should take place... at the party itself rather than before*” (A1).

### 3.3. Theme 3: Flirt Orientation

The third identified theme comprised statements regarding flirt orientation in the simulation. The current simulation is open in terms of flirt orientation because it is possible to approach characters regardless of their appearance and without influence from the chosen avatar gender. In the simulation, gender is conceptualised binarily and is represented in a scene in which a character asks the player whether they would like to approach girls or boys. This openness was noticed by participants (86,87) and was rated as “*good*” (B1, C2, D1, D2) and “*positive*” (C2). No statements against these open flirt opportunities were made. One participant further mentioned that this format was good preparation for being approached by a person of the same gender in real life (88). Moreover, another participant found it disappointing that it was not possible to have a kissing scene with the desired same-gender character and wished for even more explicit flirt possibilities with characters of the same gender, as represented in the following statement:

*“So [for] the person I wanted to get with, there was no opportunity to kiss, but [for] the person you actually just want to make friends with immediately, after three sentences, you see the option to ‘kiss her’.”* (B1)

Another participant who claimed to belong to a “queer bubble” (i.e., a queer peer group) (C2) of friends assumed that the open flirt possibilities might appear less realistic to straight peers who have not experienced this openness before (89). Still, open flirt orientation was regarded as positive because both hetero- and homo-oriented flirting is possible. In contrast, the critique of flirt options involved the perception of flirting as being “*fast*” (C1) and “*excessive*” (C2), which might draw attention away from the learning target—namely to drink less alcohol (90,91).

## 4. Discussion

Overall, the present study revealed valuable insights into adolescents’ perceptions of gender aspects in a VR simulation for alcohol prevention. Using thematic analysis, three main themes were identified: *relevance of gender*, *tailoring options*, and *flirt orientation*. While the latter two themes focus on identity or orientation aspects, the first theme—*relevance of gender*—encompasses both. Indeed, different tailoring options for the simulation were favoured with approving or disapproving statements depending on whether the relevance of female, male, or non-binary-specific aspects was stated.

The study found a clear consensus on some gender aspects among participants: The main theme of *flirt orientation* highlighted the consensus among participants that flirting options should be possible regardless both of the chosen avatar gender and of the gender appearance of characters in the simulation. In a broader context, these adolescents’ statements might reflect a decline in open homophobia in the younger generation in Germany in relation to other groups at risk of discrimination, such as asylum seekers and Muslims [36].

Additionally, the participants’ awareness of queer and non-binary peers was raised and grew through the discussions. Despite contra-arguments, such as the allegedly small numbers of non-binary individuals and the assumption that gender is binary and based on two biological sexes, in general, the topic of gender diversity was raised and discussed in each focus group. Taking up the perception of the importance of gender diversity in identity and orientation, it would be possible to add answer options for when users are asked about the person they want to approach to flirt within the simulation. This addition could increase the number of currently available choices—“I’m into boys” or “I’m into girls”—and provide further options, such as “both” or “I don’t care about gender”. An additional opportunity mentioned by participants would be to increase diversity by including non-binary characters among the actors. In any event, gender diversity should be acknowledged when conceptualising future health interventions for this age group. However, further research is needed because little is known about LGBTQIA+ adolescents’ opinions on or preferences for gender tailoring in health interventions. Thus far, LGBTQIA+ adolescents are mostly addressed as a vulnerable group in alcohol and other drugs prevention [37], but less is known about how possible tailoring options for this group in general alcohol intervention could take place and meet their needs, especially because targeted digital alcohol prevention for this group is lacking [38]. Therefore, involving this community in gender-sensitive intervention development is warranted in order to develop or enhance alcohol-prevention programmes in a gender-sensitive manner.

Furthermore, at the beginning of the focus groups, early statements about the relevance of gender initially indicated that the topic was not important to the adolescents. This was explained by the participants—inter alia—by the modern attitude of the younger generation. However, this attitude was contrasted by many subsequent statements during the ongoing discussion that illustrated the importance of gender and gender differences in the context of adolescent drinking and partying, particularly in real life. This apparent contradiction in attitudes could reflect an effort to adhere to social desirability when discussing the topic of gender. However, this social desirability for gender to no longer play a role in society may only be superficial as unequal power relations between the genders persist. The above-mentioned contradiction became especially clear when sexual harassment was brought up and prominently discussed in most of the focus groups. Similarly, studies on gender aspects in other fields have revealed a discrepancy between the egalitarian views that participants initially state and the participants’ actual, deep-seated views, which include imbalanced gender relations [39]. In this respect as well as in accordance with methodological literature [34], the use of focus groups can be regarded as an appropriate method of generating fruitful discussion among participants. The participants in our groups expressed a strong preference to address sexual harassment in alcohol prevention. While first mentioned as a topic that affects girls, the discussions revealed that the participants indeed found it important to address sexual harassment among all youth. Future research should be conducted on whether and how sexual harassment can be addressed in alcohol prevention and specifically in VR-based interventions in this field. Similar to Guldager et al. [19] demonstrating that the results of the Danish *VR FestLab* could be further intensified by increasing peer pressure [19], we also found that intensifying sexual harassment could further strengthen the simulation.

The discussions on tailoring options revealed divergent and changing positions in the course of the focus groups. Different opinions emerged both among participants within each focus group and between focus groups. In addition, some participants also changed their views during the discussion. Neither the girls nor the boys agreed on whether or how gender is relevant and should be conceptualised in the simulation, thereby resulting in both pro- and contra-arguments on different tailoring options.

Firstly, tailoring the simulation to female and male players—as is currently done with the bathroom figures for girls and boys at the beginning of the simulation—is perceived as a common albeit non-ideal option because it does not correspond to the perceived lack of differences between genders within the subsequent scenes. Furthermore, neither the girls’ nor the boys’ statements regarding their desire to be able to identify as female or male were homogenous (intra-group heterogeneity) and thus did not result in a clear recommendation to tailor the simulation according to girls and boys, as is currently done in *Virtual LimitLab*. Apart from the common perception of the irrelevance of gender within the simulation, the gender of the avatar was found to not change the simulation much—or at least not recognisably for the participants—thereby rendering it an “empty shell” without further meaning because it does not correspond to distinguishable experiences between girls and boys. The suggestion that visible gender symbols or body parts could help to identify the avatar as female or male throughout the simulation is not supported by research, which shows that body ownership, immersion, and emotional and cognitive involvement do not improve with a more coherent virtual body or body representation [40].

Secondly, the option of adding a third, gender-neutral avatar cannot yet be regarded as recommendable as the participants’ comments on the issue were too divergent and because an adequate name for and symbol of such an avatar remains unclear. Additionally, the statements that the participants made on the topic were merely conjecture, and more research is thus needed on whether and how a third category would appeal to those who do not identify with the options of female or male. Such research also requires the inclusion of LGBTQIA+ adolescents.

Thirdly, participants mostly favoured the apparently obvious option of having no gender selection for the avatar. At the same time, this option would render it difficult to form a stronger gender identification or to design specific pathways in the simulation. As persisting power relations that come into play with the topic of gender clearly emerged in the focus groups, with sexual harassment being the most prominent, it might be important to not run the risk of being gender-blind by simply eliminating gender aspects altogether. The challenge remains to take up differences and address them without reproducing stereotypes.

The final and most promising tailoring option in the case of VR seemed to be to allow adaptation to individual characteristics to occur through the actions and choices made during the simulation itself. Thus, adaptation would occur not by choosing an identity category at the beginning of the simulation but through the actions of the users and their choices throughout the simulation. This concept is similar to that presented by Zauchner-Studnicka et al. [41], who developed a gender- and diversity-sensitive digital support application for diabetes self-management in which recommendations are generated based on the occasion or on the individual rather than on literature-based gender differences.

Taking all these tailoring options and the divergent opinions on each of them into account reveals how difficult it is to formulate clear recommendations on how to realise tailoring. Nevertheless, it can be concluded from this study that none of the gender-tailoring options were clearly favoured by all of the adolescents, and that each tailoring option has its advantages and disadvantages. Not even in the small sample of this study was there consensus on tailoring options. This finding might be a result of the controversial discussion of gender issues in general. A further possible explanation of the contradictory statements may lie in the differing concepts of gender among the participants. Most participants referred to gender in a social understanding (e.g., gender identity, expression, or roles), while others might have referred to gender under the assumption that it stems from biology and that there are only two biological sexes (e.g., chromosomes, hormones, or anatomy). This might be a result of the fact that the German term “Geschlecht” comprises both concepts, whereas the English language differentiates between “sex” and “gender” [42].

Consequently, gender sensitivity should be considered when designing and customising interventions for adolescents according to gender. This means that it is necessary to be aware of and consider differences and needs in connection with gender (e.g., sexual harassment as an important topic) and to consider gender in its complexity (e.g., orientational and identity aspects that go beyond a binary understanding of the topic) while not necessarily tailoring to only two specific genders. This notion is based on the fact that there was no consensus among the girls or the boys on whether or how gender is relevant to them in the simulation or on which possible avatar options were favoured.

While one strength of the present study lies in the insights that it produced into adolescents’ perceptions of gender aspects in a VR simulation for alcohol prevention and in the rich data that the focus groups yielded in relation to the research question, several limitations must be mentioned. Firstly, as a qualitative study, our results are limited to the specific study sample and context and are thus not generalisable. The sample size is limited due to the exploratory nature of a first testing of *Virtual LimitLab.* Two gender homogenous groups for girls and boys were planned, but due to the spontaneous cancelation of participants, only one focus group with boys could be conducted. This resulted in unequal gender distribution among the sample, limiting the extent to which the opinions of male adolescents could be captured. However, since the trade-off between the number of focus groups on the one side and richness of the data and analysis on the other side is more important for the quality of qualitative studies than sample size [43], this study still provides valuable insights. A second limitation is that our sample was a convenience one and also geographically limited to Berlin to assure possible in-person participation. While data saturation was not targeted as a criterion for the sample size in this study, the applied sampling strategy is prone to self-selection bias [44]. This bias also applies to the present study, as most of the participants were attending “Gymnasium”, the highest level of secondary education in Germany. Therefore, our results must be discussed in light of these limitations, and our suggestions for further research are based on important insight but not on generalisable findings. Thirdly, although focus groups led to interaction and despite the fact that opposing opinions were given among our groups, this method is influenced by social desirability, especially among adolescents [45]. While group dynamics were used to induce discussion, marginalised opinions or positions might not have been expressed. Fourthly, neither the report nor the transcripts were returned to the participants for comments or correction. Therefore, communicative validation [30] was not possible, as misunderstandings could not be ruled out and feedback could not be captured.

## 5. Conclusions

In summary, the present study identified divergent perceptions of the *relevance of gender* (first main theme) and differing opinions on gender-*tailoring options* (second main theme) within a VR simulation for alcohol prevention. While open *flirt orientation* (third main theme) towards both the same and different genders was rated via consensus as adequate, gender-identity aspects and tailoring options for avatar gender were controversial. The prominent discussion of sexual harassment highlights the importance of addressing sexual education and alcohol prevention together in health interventions. Furthermore, results indicate that gender-sensitive research is required to capture different persisting needs while also applying a complex understanding of gender that goes beyond the binarity of male and female as fixed and clearly distinguishable entities.

## Figures and Tables

**Table 1:** Participants’ characteristics (n = 13).

Age	Gender	Type of School	Experience with Drinking Alcohol	Experience with Virtual Reality
15–18 years	1 boys’ focus group Group B, n = 4	Gymnasium, n = 11	Yes: 12 participants	Yes: 10 participants
Mean: 16 SD ^1^: 1.3	3 girls’ focus groups Groups A, n = 4 Group C, n = 2 Group D, n = 3	Gesamtschule, n = 2	No: 1 participant	No: 3 participants

^1^ SD = standard deviation.

## Data Availability

Data are not publicly available due to data protection.

## Supplementary Materials

The following supplementary materials are available online at https://www.mdpi.com/article/10.3390/ijerph19095265/s1: Table S1: Further example quotes from the dataset.

## References

[1] ESPAD Group (2020). ESPAD Report 2019: Results from the European School Survey Project on Alcohol and other Drugs.

[2] Inchley J., Currie D., Vieno A., Torsheim T., Ferreira-Borges C., Weber M.M. (2018). Adolescent Alcohol-Related Behaviours: Trends and Inequalities in the WHO European Region, 2002–2014.

[3] Kuntz B., Lange C., Lampert T. (2015). Alkoholkonsum bei Jugendlichen—Aktuelle Ergebnisse und Trends. Gesundh. Kompakt.

[4] Orth B., Merkel C. (2020). Die Drogenaffinität Jugendlicher in der Bundesrepublik Deutschland 2019. Rauchen, Alkoholkonsum und Konsum illegaler Drogen: Aktuelle Verbreitung und Trends.

[5] Dir A.L., Bell R.L., Adams Z.W., Hulvershorn L.A. (2017). Gender Differences in Risk Factors for Adolescent Binge Drinking and Implications for Intervention and Prevention. Front. Psychiatry.

[6] Jovičić Burić D., Muslić L., Krašić S., Markelić M., Pejnović Franelić I., Musić Milanović S. (2021). Gender Differences in the Prediction of Alcohol Intoxication among Adolescents. Subst. Use Misuse.

[7] Schulte M.T., Ramo D., Brown S.A. (2009). Gender differences in factors influencing alcohol use and drinking progression among adolescents. Clin. Psychol. Rev..

[8] Smit E.S., Linn A.J., van Weert J.C.M. (2015). Taking online computer-tailoring forward: The potential of tailoring the message frame and delivery mode of online health behaviour change interventions. Eur. Health Psychol..

[9] Lin H.T., Li Y.I., Hu W.P., Huang C.C., Du Y.C. (2019). A Scoping Review of the Efficacy of Virtual Reality and Exergaming on Patients of Musculoskeletal System Disorder. J. Clin. Med..

[10] Hu-Au E., Lee J. (2018). Virtual reality in education: A tool for learning in the experience age. Int. J. Innov. Educ..

[11] Durl J., Dietrich T., Pang B., Potter L.-E., Carter L. (2018). Utilising virtual reality in alcohol studies: A systematic review. Health Educ. J..

[12] Sharkey T., Whatnall M.C., Hutchesson M.J., Haslam R., Bezzina A., Collins C.E., Ashton L.M. (2020). Effectiveness of gender-targeted versus gender-neutral interventions aimed at improving dietary intake, physical activity and/or overweight/obesity in young adults (aged 17–35 years): A systematic review and meta-analysis. Nutr. J..

[13] Regitz-Zagrosek V. (2012). Sex and gender differences in health. Science & Society Series on Sex and Science. EMBO Rep..

[14] Östlin P., Eckermann E., Mishra U.S., Nkowane M., Wallstam E. (2006). Gender and health promotion: A multisectoral policy approach. Health Promot. Int..

[15] Prediger C., Helmer S.M., Hrynyschyn R., Stock C. (2021). Virtual Reality-Based Alcohol Prevention in Adolescents: A Systematic Review. Adolescents.

[16] Dietrich T., Rundle-Thiele S., Kubacki K., Durl J., Gullo M., Arli D., Connor J. (2019). Virtual reality in social marketing: A process evaluation. Mark Intell. Plan.

[17] Vallentin-Holbech L., Dalgaard Guldager J., Dietrich T., Rundle-Thiele S., Majgaard G., Lyk P., Stock C. (2020). Co-Creating a Virtual Alcohol Prevention Simulation with Young People. Int. J. Environ. Res. Public Health.

[18] Durl J., Trischler J., Dietrich T. (2017). Co-designing with young consumers—Reflections, challenges and benefits. Young Consum..

[19] Guldager J.D., Kjær S.L., Lyk P., Dietrich T., Rundle-Thiele S., Majgaard G., Stock C. (2020). User experiences with a virtual alcohol prevention simulation for danish adolescents. Int. J. Environ. Res. Public Health.

[20] Guldager J.D., Kjær S.L., Grittner U., Stock C. (2022). Efficacy of the Virtual Reality Intervention *VR FestLab* on Alcohol Refusal Self-Efficacy: A Cluster-Randomized Controlled Trial. Int. J. Environ. Res. Public Health.

[21] Urban M. (2021). Geschlechtersensible Gestaltung digitaler Gesundheitsförderung. Prävention Gesundh..

[22] Cecchini M. (2019). Reinforcing and Reproducing Stereotypes? Ethical Considerations When Doing Research on Stereotypes and Stereotyped Reasoning. Societies.

[23] Bowleg L. (2021). Evolving Intersectionality Within Public Health: From Analysis to Action. Am. J. Public Health.

[24] McCall L. (2005). The Complexity of Intersectionality. Signs.

[25] Nielsen M.W., Stefanick M.L., Peragine D., Neilands T.B., Ioannidis J.P.A., Pilote L., Prochaska J.J., Cullen M.R., Einstein G., Klinge I. (2021). Gender-related variables for health research. Biol. Sex Differ..

[26] Johnson J.L., Repta R., Oliffe J.L., Greaves L. (2012). Sex and Gender: Beyond the Binaries. Designing and Conducting Gender, Sex, & Health Research.

[27] Döring N., Bortz J. (2016). Forschungsmethoden und Evaluation in den Sozial—Und Humanwissenschaften.

[28] Schulz M., Schulz M., Mack B., Renn O. (2012). Quick and easy!? Fokusgruppen in der angewandten Sozialwissenschaft. Fokusgruppen in der Empirischen Sozialwissenschaft: Von der Konzeption bis zur Auswertung.

[29] Braun V., Clarke V. (2006). Using thematic analysis in psychology. Qual. Res. Psychol.

[30] Steinke I., Flick U., von Kardorff E., Steinke I. (2004). Quality criteria in qualitative research. A Companion to Qualitative Research.

[31] Tong A., Sainsbury P., Craig J. (2007). Consolidated criteria for reporting qualitative research (COREQ): A 32-item checklist for interviews and focus groups. Int. J. Qual Health Care.

[32] Schwan S., Buder J. (2006). Virtuelle Realität und E-Learning. https://www.e-teaching.org/didaktik/gestaltung/vr/vr.pdf.

[33] Lyk P., Majgaard G., Vallentin-Holbech L., Dalgaard Guldager J., Dietrich T., Rundle-Thiele S., Stock C. (2020). Co-Designing and Learning in Virtual Reality. Development of Tool for Alcohol Resistance Training. Electron. J. E-Learn..

[34] Daley A.M. (2013). Adolescent-Friendly Remedies for the Challenges of Focus Group Research. West J. Nurs. Res..

[35] Helfferich C. (2009). Die Qualität Qualitativer Daten. Manual für die Durchführung Qualitativer Interviews.

[36] Küpper B., Klocke U., Hoffmann L.-C. (2017). Einstellungen Gegenüber Lesbischen, Schwulen und Bisexuellen Menschen in Deutschland. Ergebnisse einer Bevölkerungsrepräsentativen Umfrage.

[37] Pienaar K., Murphy D.A., Race K., Lea T. (2018). Problematising LGBTIQ drug use, governing sexuality and gender: A critical analysis of LGBTIQ health policy in Australia. Int. J. Drug Policy.

[38] Gilbey D., Morgan H., Lin A., Perry Y. (2020). Effectiveness, Acceptability, and Feasibility of Digital Health Interventions for LGBTIQ+ Young People: Systematic Review. J. Med. Internet Res..

[39] Koppetsch C., Speck S., Behnke C., Lengersdorf D., Scholz S. (2014). Wenn der Mann kein Ernährer mehr ist. Wissen-Methode-Geschlecht: Erfassen des fraglos Gegebenen.

[40] Lugrin J.L., Ertl M., Krop P., Klüpfel R., Stierstorfer S., Weisz B., Rück M., Schmitt J., Schmidt N., Latoschik M.E. Any “Body” There? Avatar Visibility Effects in a Virtual Reality Game. Proceedings of the IEEE Conference on Virtual Reality and 3D User Interfaces (VR).

[41] Zauchner-Studnicka S., Hornung-Prähauser V., Plößnig M., Leutner M., Kautzky-Willer A. (2016). Wie Gender in die Diabetes-Selbstmanagement-Applikation kommt—Ein vielversprechender Weg [How gender is included in a diabetes self-management application—A promising approach]. GENDER Z. Geschlecht Kult. Ges..

[42] Villa P.-I., Kortendiek B., Riegraf B., Sabisch K. (2018). Sex—Gender: Ko-Konstitution statt Entgegensetzung. Handbuch Interdisziplinäre Geschlechterforschung.

[43] Carlsen B., Glenton C. (2011). What about N? A methodological study of sample-size reporting in focus group studies. BMC Med. Res. Methodol..

[44] Etikan I., Abubakar Musa S., Alkassim R.S. (2015). Comparison of Convenience Sampling and Purposive Sampling. Am. J. Appl. Stat.

[45] Adler K., Salanterä S., Zumstein-Shaha M. (2019). Focus Group Interviews in Child, Youth, and Parent Research: An Integrative Literature Review. Int. J. Qual..

