# Supplementary material for: Adolescents’ Perceptions of Gender Aspects in a Virtual-Reality-Based Alcohol-Prevention Tool: A Focus Group Study
Source: Int J Environ Res Public Health. 2022 Apr 26;19(9):5265. doi: 10.3390/ijerph19095265 (PMC9100656; doi:10.3390/ijerph19095265)
Supplement: Supplementary file 1 [file ijerph-19-05265-s001.zip › ijerph-1658650-supplementary.pdf]

**Adolescents' Perceptions of Gender Aspects in a Virtual-Reality-Based Alcohol-Prevention Tool: A Focus Group Study**

Table S1: Further example quotes from the dataset

| No. | Quote translation (participant) | Quote German original (participant) |
|---|---|---|
| 1. | The relevance is not sooo... Well... you wouldn't think that you have the feeling that something is missing somehow if you leave it out. You wouldn't miss anything big then. (A1) | Die Relevanz ist jetzt nicht sooo... Also... man würde sich jetzt nicht denken, dass man das Gefühl hat, dass irgendwie etwas fehlt, wenn man es weglässt. Man würde dann nichts groß vermissen. (A1) |
| 2. | So, I believe there [in the simulation] doesn't have to be a [gender] difference. (D3) | Also ich glaube da [in der Simulation] muss kein Unterschied [nach Geschlecht] sein. (D3) |
| 3. | Let me put it this way, there were no such differences, where you´d say 'Yes, now I've noticed that I've somehow... played a woman. ' (A2) | Sag' ich mal so, es gab jetzt keine krassen Unterschiede, wo man jetzt so sagt ´Ja, also jetzt hab' ich gemerkt, dass ich irgendwie... eine Frau gespielt hab.'(A2) |
| 4. | It is just not noticeable whether you have decided to be male or female. (A3) | Es fällt halt auch nicht auf, ob man sich jetzt für männlich oder weiblich entschieden hat. (A3) |
| 5. | But to be honest, I didn't notice such a difference (A2). | Aber ich hab' ehrlich gesagt jetzt nicht so 'nen krassen Unterschied gemerkt. (A2) |
| 6. | It's like, you do choose it, but then you forget. (B2) | Es ist auch so, man wählt es zwar aus, aber dann hat man's wieder vergessen. (B2) |
| 7. | With me, I forgot it for a moment. (D1) | Bei mir, hab' ich's kurz vergessen. (D1) |
| 8. | Because you just don't see yourself. So, I would rather leave it [gender] out. (B1) | Weil du halt dich selber halt nicht siehst. Also ich würde es [Geschlecht] eher so weglassen. (B1) |
| 9. | I think because you don't see yourself anyway. (D1) | Ich find' weil du siehst dich ja selbst eh nicht. (D1) |
| 10. | You´ve not been addressed as he or she... (C1) | Man wurde auch nicht mit er oder sie... (C1) |
| 11. | ...Always was used a "You" and that could be a boy or a girl. That was just... not really relevant... (C1) | ...immer geduzt und das könnte ein Junge oder ein Mädchen sein. Das war halt... nicht wirklich relevant... (C1) |
| 12. | Yes, I think for our generation it [the gender difference] is not so very different now. So, whether you are a boy or a girl when you go to a party. (B4) | Ja, ich glaub' für unsere Generation ist es [Geschlechterunterschiede] jetzt nicht so ganz groß anders. Also ob man jetzt ein Junge oder ein Mädchen ist und dann auf 'ne Party geht. (B4) |
| 13. | I think personalisation should take place... in the event of the party itself, rather than before... before the party itself. Because all those things can't really have much influence on the party. But if you choose, for example, which drinks you drink, whether you drink a Coke or a Fanta or a Sprite or I don't know... alcohol or stuff. Something that somehow has more to do with the party. (A1) | Also ich find Personalisierung sollte eher so... im Geschehen der Party selbst stattfinden, als davor... vor der Party an sich. Weil die ganzen Sachen, einfach wenig Einfluss auf die Party eigentlich haben können. Aber wenn man sich zum Beispiel aussucht welche Getränke man trinkt, ob man 'ne Cola, oder 'ne Fanta oder 'ne Sprite oder keine Ahnung...Alkohol halt oder Zeugs halt. Etwas das irgendwie noch etwas genauer mit der Party zu tun hat. (A1) |
| 14. | I think it's important who you are right now. (D1) | Ich finde es ist wichtig, wer du gerade bist. (D1) |

| | | |
|---|---|---|
| 15. | Yes, yes, because then you also speak differently. (D3) | Ja, ja, weil du dann auch anders sprichst. (D3) |
| 16. | Yes, because you speak differently. Well... the way people talk to me, well... people talk differently. I talk differently. […] You can tell the difference... I don't know... when flirting or something, that you also notice, I am the girl or I am the boy, yes. (D1) | Ja, weil du dann auch anders sprichst. Na ja... wie man mit mir redet, also so... man redet ja anders. Ich red' anders. […] Einfach dass man den Unterschied merkt, so... keine Ahnung... jetzt beim Flirten oder so was, dass man auch merkt, ich bin gerade das Mädchen oder ich bin gerade der Junge, ja. (D1) |
| 17. | So, I think it kind of brings a change in real life. (C2) | Also ich glaube, dass es irgendwie in Real Life eine Veränderung mit sich bringt. (C2) |
| 18. | I've noticed that men tend to be more aggressive, when I'm drinking or… with my head somewhere else… they are much more offensive, hit more below the belt, when it comes to flirting. I would also add more of that [in the simulation]). (A1) | Ich hab´ eher die Erfahrung gemacht, dass Männer offensiver rangehen, wenn sie merken dass ich getrunken hab' oder so, oder dass ich jetzt grade... ein bisschen mit mein Kopf woanders bin... gehen sie einfach viel offensiver, viel... mehr unter die Gürtel sag' ich mal so... mit dem Flirten auch. Das würde ich auch ein bisschen reinmachen [in die Simulation]. (A1) |
| 19. | It's just still the case that women have to pay much more attention at a party (B3) | Aber es ist halt doch immer noch der Fall, dass Frauen auf 'ner Party auch deutlich mehr achten müssen. (B3) |
| 20. | It is also factually the case that men are at a lower risk for such things. (B3) | Es ist ja auch faktisch so, dass bei Männern die Gefahr für solche Sachen niedriger ist. (B3) |
| 21. | There are men who simply get women drunk in order to get something from them afterwards, and I think that as a man you really don't have to worry so much about that. But as a woman, I think you do, yes. In this setting, it is not so important, but it is relevant for women I think, and is definitely part of alcohol prevention. (B2) | Es gibt halt Männer, die Frauen einfach abfüllen, um danach im Nachhinein von denen was zu bekommen und ich find' man muss sich als Mann sich wirklich nicht so viel Sorgen darüber machen. Aber als Frau glaub ich schon, ja. In dem Setting ist es nicht so wichtig, aber es ist relevant für Frauen find' ich und gehört bei der Alkoholprävention auf jeden Fall dazu. (B2) |
| 22. | At my age, it would be important to be able to see what can happen if someone hits on you and you are not sane, what can happen. So... in general, as far as sexual harassment is concerned, I think that should be included maybe because people also act differently when they are under the influence of drugs, or here alcohol, and then.... it can be included. (A1) | In mein Alter wäre es schon wichtig, dass man auch sehen kann, was kann passieren, wenn jemand dich anmacht und du bist grade nicht zurechnungsfähig, was kann passieren. Also... allgemein was so sexuelle Belästigung angeht, ich finde das sollte man vielleicht einbeziehen, weil Menschen ja auch anders handeln, wenn sie unter Einfluss von Drogen, oder jetzt Alkohol sind und dann... kann man das ruhig auch einbeziehen. (A1) |
| 23. | It's relevant for women, I think, and definitely belongs in alcohol prevention. (B2) | Es ist relevant für Frauen, find' ich, und gehört bei der Alkoholprävention auf jeden Fall dazu. (B2) |
| 24. | Of course, men are also being approached, but it's more realistic, I think. If for me... if a girl would make a pass at a boy like that, I'm sure there are some girls that would do that, I don't want to exclude it, but I don't think it would be realistic (D2). | Also natürlich, werden auch Männer angequatscht, aber es ist einfach realistischer, find' ich. Also würde mir da jetzt, würde ein Mädchen sich so an Jungen ranmachen, gibt es bestimmt, will ich nicht ausschließen, aber ich find's nicht realistisch. (D2) |

| | | |
|---|---|---|
| 25. | A4: You could do it like that, that you can choose your clothes, for example, if you choose very skimpy clothes, you would get more harassment, but that is, of course, more complicated to do.<br>A3: But that is... that is exactly the motto 'Oh, she was wearing a short skirt!'<br>A4: Yeah... I know...<br>A3: 'She wanted to be touched.... ' and that's wrong!<br>A1: Yeah, it's kind of the wrong message. That if you wear skimpy clothes then you're more likely to be harassed. I would rather show the message that it can always happen…<br>A3: Yes, that's also true.<br>A1: And that´s why you should wear what you want. | A4: Man könnte mal natürlich auch so machen, dass man sich die Klamotten aussucht und zum Beispiel, wenn man so ganz knappe Klamotten sich dafür aussucht, dass dann mehr Belästigung bekommt, aber so was ist dann natürlich schon komplizierter zu machen.<br>A3: Aber das ist... das ist genau unter dem Motto ´Oh, sie hatte einen kurzen Rock an!´<br>A4: Ja... ich weiß... (A4)<br>A3: ´Sie wollte angefasst werden... ´ und das ist falsch!<br>A1: Ja, es ist irgendwie die falsche Message. Das, wenn du knappe Klamotten trägst, dann wirst du eher belästigt. Ich würde eher die Message zeigen, dass es immer passieren kann...<br>A3: Ja, das stimmt auch.<br>A1: Und dass du deshalb tragen sollst, was du willst. |
| 26. | It still depends on which target group you choose, let's say you want tenth graders or something... difficult! (A1) | Es kommt trotzdem drauf an, welche Zielgruppe man wählt, sagen wir mal ihr wollt Zehntklässler oder so... schwierig! (A1) |
| 27. | Of course, sexual harassment is not something you should concentrate on in the game, so... let's say it was like this, [...] the game is actually about alcohol consumption and to bring that closer to the younger ones, then you should really concentrate more on that. (B1) | Klar ist dann halt, sexuelle Belästigung, nicht das worauf man sich in dem Spiel konzentrieren sollte, also... sagen's mal so, es war so, […] das Spiel geht halt eigentlich auch nur um den Alkoholkonsum und das halt den Jüngeren nahezubringen, dann sollte man sich wirklich mehr darauf konzentrieren. (B1) |
| 28. | But first of all, I don't know how useful that would have been on a... in the simulation and to what extent it would have been possible, so... (C2) | Ich weiß aber erstens nicht wie sinnvoll das gewesen wär' das auf einer... in der Simulation nochmal mehr darzustellen und inwiefern es auch gegangen wär', also... (C2) |
| 29. | But I don't think that you should specialize it [harassment in the simulation] one gender, because you know that it doesn't only happen to one gender. (A1) | Aber ich find' nicht, dass man das [Belästigung in der Simulation] dann jetzt auf ein Geschlecht spezialisieren soll, weil man weiß ja, dass es nicht nur bei einem Geschlecht vorkommt. (A1) |
| 30. | I have changed my mind. I think if so, then it [the portrayal of sexual harassment in the simulation] should not be connected to a certain gender" (A2). | Ich hab' meine Meinung geändert. Ich finde wenn, dann man sollte es [Darstellung sexueller Belästigung in der Simulation]schon gar nicht auf's Geschlecht irgendwie bezogen werden, was da alles passieren kann. (A2) |
| 31. | Maybe when a girl speaks to me and says, "Hey, what do you think of that boy?" Something like... that the girl arrives and so like girls talk at a party. So ... Yes, so, then a girl comes, she says 'Hey hey blablabla', short small talk and then she says 'Um, I noticed, the guy is looking at you all the time. Try it with him.' Kind of like that. (D1) | Wenn vielleicht ein Mädchen mich anspricht und dann so sagt ´Ey, wie findest du den Jungen?´ So was... das so das Mädchen ankommt und so, wie Mädchen halt auf 'ne Party reden so. Also... ja, also, dann kommt so ein Mädchen, sie sagt ´Hey hey blablabla´ kurz Smalltalk und dann sagt sie so ´Ehm, mir ist aufgefallen, der Typ start dich die ganze Zeit so an. Versuch's bei ihn.´ Irgendwie so was." (D1) |

| | | |
|---|---|---|
| 32. | Maybe you can make a little symbol up there. With this you could assess very well, am I now 'a boy' or am I a girl? (D2) | Vielleicht kann man da oben so ein kleines Zeichen machen. Damit könnte man sehr gut einschätzen, Bin ich jetzt 'nen Junge oder bin ich ein Mädchen? (D2) |
| 33. | Just girls have high heels and a skirt on and the boy just has jeans on. (D1) | Einfach Mädchen haben jetzt High Heels und ein Rock an und der Junge hat einfach nur Jeans an. (D1) |
| 34. | Or, for example, if you're a boy and you walk up to a boy, then... so, if you're a boy, he would say, for example, 'Hey, bro, what's up?` And if you're a girl, maybe something like, 'You look really good` Something like that. (D3) | Oder so zum Beispiel, wenn du grad 'nen Junge bist und du auf 'nen Jungen zugehst, dann... so, wenn du ein Junge bist, würde er zum Beispiel so sagen ´Ey, Bro, was geht?` Und wenn du ein Mädchen bist, vielleicht irgendwie so ´Du siehst voll gut aus.` So etwas halt. (D3) |
| 35. | D1: When you approached a boy as a boy, he was like, 'Hey, bro. I'm not gay.'<br>D2: Of course, it's okay if he didn't want to, but... that's just not how you react. But I thought it was good that he said that, because it was so... of course not good, I don't know, it was totally mean, but... in the... good from a game point of view. | D1: Als man als Junge ein Junge angesprochen hat dann meinte der so, ´Ey, ne Bro. Ich bin nicht schwul.´<br>D2: Natürlich ist es ok, wenn er es nicht wollte, aber... so reagiert halt man nicht. Aber das fand ich gut, dass er das gesagt hat, weil es war so... natürlich nicht gut, keine Ahnung, es war voll gemein, aber... in dem... vom Spiel her gut. |
| 36. | Well, personally, I don't need it [a non-binary avatar]. My flatmate is a trans person and there was a non-binary person in my school [...] If you want to reach everyone, you should try to reach everyone. (A3) | Also ich persönlich für mich brauch's [a non-binary avatar] nicht. Mein Mitbewohner ist ein Transmensch und in meiner Schule war eine non-binary Person […] Wenn man alle erreichen möchte, sollte man ja versuchen alle zu erreichen. (A3) |
| 37. | I think there are people who do have a problem with that, for whom that [a binary choice] is less realistic. (C2) | Ich denke, dass es Leute gibt, die damit schon ein Problem haben, für die das [eine binäre Auswahl] weniger realistisch ist. (C2) |
| 38. | And the one who feels different, the one who doesn't feel masculine or feminine, who plays that, can choose. (D2) | Und der, der sich dann anders fühlt, also der der sich nicht als männlich oder weiblich fühlt, der das spielt, kann das ja auswählen. (D2) |
| 39. | On the other hand, yes... I think in a party situation, you would quite quickly experience that you are simply pigeonholed. (B2) | Auf der anderen Seite ja... ich glaub' in einer Partysituation, wurde man ziemlich schnell die Erfahrung machen, dass man einfach eingeordnet wird. (B2) |
| 40. | Yes, so I don't know, I don't think that would cover society in a different way if you... what is the proportion of non-male or non-female people in society? That's a fraction of a fraction (B3). | Ja, also keine Ahnung, ich finde das würde jetzt nicht groß die Gesellschaft anders abdecken, wenn man jetzt... was ist der Anteil von nicht-männlichen oder nicht-weiblichen Personen in der Gesellschaft? Das ist so 'nen Bruchteil von Bruchteil (B3) |
| 41. | I... didn't have a problem with the selection or anything like that, it wasn't a problem for me. (A2) | Ich... hatte jetzt damit auch kein Problem mit der Auswahl oder so, für mich war's auch kein Problem. (A2) |
| 42. | Well, I have to say, I didn't really think about the options of taking a male game character. Because I thought, ok, I'll take what I... um... identify with at the moment. Something like that, um. (C2) | Also ich muss sagen, ich hab' gar nicht an sich über die Optionen nachgedacht, 'ne männliche Spielerfigur zu nehmen. Weil ich dachte, ok ich nehme das was ich... ehm... womit ich mich zur Zeit identifiziere. So ungefähr, ehm. (C2) |

| | | |
|---|---|---|
| 43. | I think then you also have the problem that as a man you can no longer identify with it very much, or as a woman. (B2) | Ich glaube dann hat man auch das Problem, dass man als Mann sich nicht mehr damit sehr identifizieren kann, oder halt als Frau. (B2) |
| 44. | Yes, I think it's [the binary choice] good, because you can choose whether you want to be male or female and then... yes, I don't know, if you feel somehow different, you can choose, how someone feels, when you... or how you come across, so that you can always choose how you feel like and it's not, so to speak, predetermined. (B4) | Ja, also ich find's [die binäre Auswahl] halt gut, weil man kann halt auswählen, ob man männlich oder weiblich sein will und dann... ja, halt selber weiß nicht, wenn man sich irgendwie anders fühlt, kann man das (?) wie jemand sich fühlt, wenn man... oder halt wie man rüberkommt, also dass man halt dann immer auswählen kann, wie man grade Lust drauf hat und es nicht sozusagen vorgegeben ist.(B4) |
| 45. | There are 29 genders or what. (D1) | Da stehen da halt 29 Geschlechter oder was. (D1) |
| 46. | I think it's just that you don't notice it so quickly, if you feel that you are being addressed, then you don't notice so quickly that others might not have this option. (A4) | Ich glaub nur man merkt's nicht so schnell, wenn man selbst halt sich angesprochen fühlt, dann merkt man nicht so schnell, dass andere vielleicht nicht diese Option haben. (A4) |
| 47. | Otherwise, you are not really addressed as a man or a woman in the game, rather it remains relatively gender-neutral, no matter what you choose. (B1) | Ansonsten wirst du im Spiel nicht wirklich als Mann oder Frau angesprochen, sonders es bleibt relativ geschlechtsneutral, egal was du auswählst. (B1) |
| 48. | Let's put it this way, if I had the choice between tailored and open, I would choose the open version and then not be further defined myself, so to speak. (C2) | Sagen wir's mal so, wenn ich die Wahl hätte zwischen zugeschnitten und offen, würde ich die offene Version wählen und dann sozusagen selber nicht weiter definiert sein. (C2) |
| 49. | And if you don't have a gender and are addressed by both sides, then it is... best, actually. (A4) | Und wenn man dann eh kein Gender hat und von beide Seiten angesprochen wird ist es ja dann... am besten eigentlich. (A4) |
| 50. | Then it would actually be... It would be best not to do anything at all, but to go straight in so that it starts directly without gender really playing a role. (C1) | Dann wäre es halt eigentlich auch... am besten, dass man gar nicht was macht, dass man (?), sondern dass man direkt so reingeht, dass es direkt anfängt oder dass halt Geschlecht wirklich 'ne Rolle spielt. (C1) |
| 51. | After all, it wouldn't be noticed that you can't chose. (A3) | Es würde doch nicht auffallen, dass man nicht wählen kann. (A3) |
| 52. | So you could just get rid of the gender and it wouldn't change the game that much. (B2) | Also man könnte es einfach das Geschlecht losmachen und es würde sich am Spiel nicht so viel ändern. (B2) |
| 53. | So I don't think, if the changes are not serious, I don't think this player selection was fundamentally important. (C2) | Also ich denke nicht, wenn die Änderungen nicht gravierend sind, dann denk' ich nicht, dass diese Spielerauswahl grundlegend wichtig war. (C2) |
| 54. | I think that no choice is more pleasant for such people than either a double or triple choice, where you have girl, boy, non-binary. I think just not having a choice is more comfortable. (C2) | Ich glaube, dass keine Auswahl für solche Personen angenehmer ist, als eine entweder eine zweifache beziehungsweise dreifache Auswahl, wo man dann, Mädchen, Junge, non-binary hat. Ich denke mal eine einfach nicht vorhandene Auswahlmöglichkeit ist angenehmer. (C2) |

| | | |
|---|---|---|
| 55. | Yes! I also think it's a very good idea, without gender! That's also... that could be criticised… this game now uses gender, especially at the beginning. I would just leave it out completely. (A2) | Ja! Finde ich auch eine sehr gute Idee, ohne Gender! Das ist auch... kann darauf... das könnte in Kritik kommen...Ja, dieses Spiel nutzt jetzt extra am Anfang noch Gender würde ich es einfach komplett raus lassen. (A2) |
| 56. | I think this option is good, that you go like this and then, but then you just have to click on that and not an extra game [for non-binaries]. (D2) | Ich finde diese Option gut, dass du auch so hingehst und dann, aber dann musst du das halt anklicken und nicht ein extra Spiel [für nicht-binäre]. (D2) |
| 57. | I think you could add it [a third option]. (B2) | Ich find' man könnte es [eine dritte Option] hinzufügen. (B2) |
| 58. | I don't think it's absolutely necessary [a third option]. (D3) | Finde ich auch nicht unbedingt nötig [eine dritte Option]. (D3) |
| 59. | I actually think it would be counterproductive, so... I don't think it has much to do with acceptance in society, but so... I think there are just two genders, biologically. (B3) | Ich hielte das tatsächlich für kontraproduktiv, also... ich denke es hat nicht groß irgendwie mit Akzeptanz in der Gesellschaft zu tun, also, sondern so... ich denke es gibt halt zwei Geschlechter, biologisch. (B3) |
| 60. | The character of a party is realistic that you are quickly assigned to a gender. [...] um and yes, well... I don't see any need for a different gender because as long as you don't put a sign on saying ´I'm non-binary´, you're either classified as male or female, at least by most people, so... (B3 | Die Eigenschaften von einer Party ist, dass man ziemlich schnell eben in ein Geschlecht eingeordnet wird. […] ehm und ja, also... ich sehe da keine Not für anderes Geschlecht weil so lange man sich nicht irgendwie ein Schild dranhängt mit Ich bin nonbinary. wird man entweder als männlich oder weiblich eingestuft, von den meisten Leuten zumindest, also. (B3) |
| 61. | But it doesn't have to be the... the gender you choose doesn't have to be the gender you feel to be. It's about what you want to play and if someone is like that and says 'No, I really want to be diverse now', well... I don't know. (D1) | Aber, es muss ja nicht sein die... das Geschlecht, was du auswählst, muss ja nicht das Geschlecht sein, wo du dicht fühlst. So, ehm... es geht ja darum was du spielen möchtest und wenn halt jemand jetzt so ist und sagt Ne, ich will jetzt unbedingt divers sein. Ja, also... ich weiß jetzt nicht. (D1) |
| 62. | If you introduce a third option, that it then remains the same as with the other two. But then you can just leave it [the gender choice] out. (B4) | Wenn man eine dritte Option einführt, dass es dann gleichbleibt so wie bei den anderen zwei. Aber dann kann man es [die Geschlechtsauswahl] gleich rauslasen. (B4) |
| 63. | D2: But there... there are certainly people...<br>D1: Who feel offended.<br>D2: Who are so sensitive that they don't like it when there are only two [gender options]. | D2: Aber es... gibt bestimmt Leute...<br>D1: Die sich angegriffen fühlen.<br>D2: Die so sensibel sind, die das gar nicht gut finden, wenn's nur zwei [Geschlechtsoptionen] gibt. |
| 64. | Personally, I would say that if the game were English-speaking, I would add the third option, non-binary. Then it would just be normal to address as they/them. That would just be easier in English. Just because there's no real term in German and many people are still wondering... I'd say I'd wait until it's fully developed in Germany. So that there really is an official term for it. (B1) | Also ich persönlich würde es sagen, wäre das Spiel im englischsprachigen Bereich, würde ich die dritte Option hinzufügen, halt non-binary. Dann wäre halt für die normale Ansprache they/them. das wär´ halt im Englisch einfach einfacher. Nur weil es im Deutsch kein wirklicher Begriff gibt und viele sich noch stutzig sind… Ich würd' mal sagen, damit würd' ich mal warten bis es in Deutschland ausgereift ist. Damit es wirklich ein offizieller Begriff dafür gibt, ansonsten wirst du im Spiel |

| | | |
|---|---|---|
| | | nicht wirklich als Mann oder Frau angesprochen, sondern es bleibt relativ geschlechtsneutral, egal was du auswählst. (B1) |
| 65. | Something like that would be cool. You can just choose what you look like and then of course everyone can do it individually. (A4) | So was wär auch cool. Kannst du dir halt aussuchen wie du aussiehst und dann kann natürlich jeder das auch individuell machen. (A4) |
| 66. | That would also distract a bit from the fact that... somehow from the topic of prevention and alcohol and so on, so.... (B3) | Das würde auch ein bisschen davon ablenken, dass...irgendwie von dem Thema Prävention und Alkohol und so, also... (B3) |
| 67. | I would say no because from experience I would say that it takes a long time. It's completely pointless in a game that I don't think you want to play for days. To build a memoji intensively and I... would spend a long time and lose my attention. (B2) | Ich würde sagen, nein, weil aus der Erfahrung würde ich sagen, dass das sehr lange dauert. Es ist komplett sinnfrei, in einem Spiel was man glaub ich nicht über Tage hinweg spielen möchte. Jetzt da intensiv ein Memoji zu bauen und ich... würde eine lange Zeit damit verbringen und meine Aufmerksamkeitsspanne verlieren. (B2) |
| 68. | I also think that if you can individualise the person when this does not affect what happens afterwards, then it's superfluous. (A1) | Glaub auch, dass wenn man sich die Person jetzt individualisieren kann, wenn es halt kein Effekt auf das Geschehen danach hat, dann ist es überflüssig. (A1) |
| 69. | Of course, there are also women who can tolerate much more [alcohol] than some men. (B1) | Gibt natürlich auch Frauen die, viel mehr vertragen als manche Männer. (B1) |
| 70. | I think you can get a wrong image, it can just be harmful [...] if you overestimate yourself and um... it can end very unpleasantly. (B2) | Ich glaub', man kann ein falsches Bild bekommen, es kann einfach schädlich sein, [..] wenn man sich selber überschätzt und ehm... es kann sehr unschön enden. (B2) |
| 71. | Really kind of adapted to the actual physical constitution and so on (B3). | Halt wirklich irgendwie auf die tatsächliche körperliche Konstitution und so angepasst (B3) |
| 72. | I would lie about my weight. (A4) | Ich würd bei mein Gewicht lügen. (A4) |
| 73. | [Referring to the peer group] We are 8 people, 5 people have problems with their weight... and to ask about the weight is… well ok, then you have a more personal experience, so maybe it's better to calculate what you really tolerate... but half would enter something lighter and the other half doesn't know their weight. (A3) | [Bezug auf peer group] Wir sind 8 Menschen, davon haben 5 Menschen Probleme mit ihrem Gewicht... und nach der Gewicht zu fragen ist… ja ok, dann hat man eine noch persönlichere Erfahrung, also vielleicht besser ausgerechnet was du wirklich verträgst... aber die Hälfte würde irgendwas leichteres angeben und die andere Hälfe kennt sein Gewicht nicht. (A3) |
| 74. | Maybe you can make it so individualised, like, here you have the basic values, the normal average values, and then you can also make your individualised game and enter your own values. (A4) | Vielleicht kann man das ja so individualiesiert machen, von wegen, hier hast du die Basiswerte, also die normalen Durchschnittswerte und dann du kannst auch noch dein individualisiertes Spiel machen und deine eigene Werte eingeben. (A4) |
| 75. | D1: But you don't know what for.<br>D3: So then you'd need a short explanation.<br>D1: Yes. | D1: aber man weiß es ja nicht wozu.<br>D3: also dann müsste noch 'ne kurze Erklärung.<br>D1: Ja. |

| | | |
|---|---|---|
| 76. | You could maybe divide it up like this, not what gender you are, but what gender you are attracted to, maybe you could do it like that. (B3) | Man könnte das vielleicht so einteilen, nicht was man selbst für ein Geschlecht hat, sondern zu welchem Geschlecht man sich hingezogen fühlt, so könnte man es machen vielleicht. (B3) |
| 77. | Then it's exactly the problem [if you leave out gender] that 50% of the people you chat up or who chat you up are irrelevant to you. (B3) | Dann ist es genau eben das Problem [wenn man Geschlecht weg lässt] das man 50% der Leute die man anquatscht oder die einem selbst anquatschen für einen unerheblich sind. (B3) |
| 78. | So I think it's like that now, at least in my bubble (laughs)... the party guests are more diverse. There's a certain range of people who can be seen in the game, so to speak... (C2) | Also ich denke, es ist mittlerweile zumindest in meiner Bubble (lacht) so... ist die... sind die Partygäste unterschiedlicher. Halt 'ne gewisse Spannweite an Person die im Spiel zu sehen sind, sozusagen... (C2) |
| 79. | More diverse characters, would be a bit better there. (C1) | Unterschiedlichere Charaktere, wär' da ein bisschen besser. (C1) |
| 80. | At least in my circle more realistic or maybe people who speak with an accent or something like that. (C2) | zumindest in meinen Kreis realistischer oder vielleicht Menschen die mit Akzent reden oder ähnliches. (C2) |
| 81. | Or people... there are people who don't know German really or have just come here and just yes... (C1) | Ja, oder Leute... es gibt Leute, die nicht wirklich Deutsch können oder grad hergekommen sind und halt ja... (C1) |
| 82. | Maybe you could put a bit more emphasis on diversity in the appearance of the people, so maybe one or two more... um... dark-skinned people or something. (C2) | Vielleicht könnte man so ein bisschen mehr auf Diversität beim Aussehen der Menschen setzen, also vielleicht noch ein, zwei mehr... ehm... dunkelhäutige Menschen oder so was reinbringe |
| 83. | D3: There were no dark-skinned people at all.<br>D2: But it doesn't have to be racist. I don't think that people who play the game think, 'Oh, this game is so racist. There are no foreigners here or others.' I don't know how to say it properly.<br>D3: Well, I don't think it's bad, I just think from different perspectives. | D3: Es gab gar keine dunkelhäutigen Menschen.<br>D2: Es muss aber nicht rassistisch sein. Also ich glaub' nicht, dass Leute, die das spielen halt denken, `Oha, das Spiel ist so rassistisch. Hier sind keine Ausländer oder andere.. ´Eh ich weiß nicht wie ich es richtig sagen kann.<br>D3: Also ich find's jetzt nicht schlimm, ich denke jetzt nur so aus anderen Perspektiven. |
| 84. | I think personalisation should take place... in the happening of the party itself, rather than before... Because all these things can't really have much influence on the party. But if you choose, for example, which drinks you drink, more precisely whether you drink a Coke or a Fanta or a Sprite or I don't know... alcohol or stuff, that you somehow have something more to do with the party. (A1) | Also ich find Personalisierung sollte eher so... im Geschehen der Party selbst stattfinden, als davor... vor der Party an sich, weil die ganzen Sachen, einfach wenig Einfluss auf die Party eigentlich haben können, aber wenn man sich zum Beispiel aussucht... welche Getränke man trinkt, genauer ob man 'ne Cola, oder 'ne Fanta oder 'ne Sprite oder keine Ahnung...Alkohol halt oder Zeugs halt ist, dass man irgendwie noch etwas genauer mit der Party zu tun hat. (A1) |
| 85. | Yes, I think the party behaviour, whether you're more the type who just dances all the time, is somehow on the move and drinks a lot, or whether you'd rather sit down for a conversation or maybe you'd rather sit alone. That's the decisive thing. (B3) | Ja, ich denke, das Partyverhalten ob man jetzt eher der Typ ist der die ganze Zeit nur tanzt nur irgendwie im Bewegung ist und super viel trinkt, oder ob man sich lieber zu 'ner Konversation dazu setzt oder man vielleicht doch lieber alleine sitzt. Das ist doch das entschiedende. (B3) |

| | | |
|---|---|---|
| 86. | There was also the scene with Kat where you could kiss her too, that's so... it doesn't matter what gender you are. (A2) | Es gab ja auch die Szene dann mit Kat wo du dann auch sie küssen konntest oder so, dass ist so... ist ja egal, welches Geschlecht du hast. (A2) |
| 87. | I was just, or just chatted up boys and girls um... that wasn't a problem. (B3) | Ich wurde halt, oder habe Jungs und Mädchen angequatscht ehm... das war jetzt nicht das Problem. (B3) |
| 88. | So it could be a bit appropriate that maybe a girl hits on or a boy hits on, that you have both. So you can really prepare yourself for both. No idea what happens when a girl hits on you. (C1) | Also könnte schon ein bisschen passend sein, dass vielleicht mal ein Mädchen anmacht oder dass auch ein Junge jemanden anmacht, dass man beides hat. So wirklich so halt schon mal vorbereiten kann auf beides schon mal. Keine Ahnung hat, was da passiert, wenn dich ein Mädchen anmacht... (C1) |
| 89. | Well, I have to say, I'm in a pretty queer bubble anyway, so (laughs) I was less surprised. Um... I mean... I'm sure there will be people who are... surprised, who can maybe identify less with it. For me, it was no problem. I think there are people who already have a problem with it, for whom it's less realistic, but.... (C2) | Also ich muss sagen, ich bin sowieso in eine ziemlich queeren Bubble von daher (lacht) war ich da jetzt weniger verwundert. Ehm... ich meine... es wird bestimmt Leute geben, die das...wundert, die sich vielleicht damit weniger identifizieren können. Für mich war's kein Problem. Ich denke, dass es Leute gibt, die damit schon ein Problem haben, für die das weniger realistisch ist, aber... (C2) |
| 90. | So this one... I actually thought the kissing scene on the couch was quick. This 'Do you like dancing?' that's more realistic. So, um... um... Yes, I found that less exaggerated, I'll say that now. (C2) | Also diese... ich fand eigentlich die Kussszene auf der Couch schnell. Dieses ´Magst du Tanzen?´ das ist realistischer. Also... ehm... ja, das fand ich weniger überzogen, sag' ich jetzt mal. (C2) |
| 91. | I think that somehow put the whole party in a different light. I think there are certainly parties where the goal is to go home with someone else. But I don't think that has to be the case here. I think that somehow gives an impression that doesn't correspond to reality, so... I personally haven't had such parties yet... where there was a concrete... yes... (B3) | Ich finde, das hat die ganze Party irgendwie in ein anderes Licht gedrängt. Also ich finde, also es gibt sicherlich Partys wo es am Ende dann wirklich das Ziel gibt, dass man irgendwie mit einer anderen Person nach Hause geht. Aber ich finde das muss hier jetzt nicht sein. Ich finde das vermittelt auch irgendwie ein Eindruck, der nicht der Realität entspricht, also... bei mir persönlich waren es jetzt, gab's solche Partys jetzt noch nicht... wo es dann ein konkretes... ja... (B3) |
